# Compositionality in animals and humans

**DOI:** 10.1371/journal.pbio.2006425

**Published:** 2018-08-15

**Authors:** Simon W. Townsend, Sabrina Engesser, Sabine Stoll, Klaus Zuberbühler, Balthasar Bickel

**Affiliations:** 1 Department of Comparative Linguistics, University of Zurich, Zurich, Switzerland; 2 Department of Psychology, University of Warwick, Coventry, United Kingdom; 3 Psycholinguistics Laboratory, Department of Comparative Linguistics, University of Zurich, Zurich, Switzerland; 4 Institute of Biology, University of Neuchatel, Neuchatel, Switzerland; 5 School of Psychology and Neuroscience, University of St Andrews, St Andrews, United Kingdom

## Abstract

A key step in understanding the evolution of human language involves unravelling the origins of language’s syntactic structure. One approach seeks to reduce the core of syntax in humans to a single principle of recursive combination, merge, for which there is no evidence in other species. We argue for an alternative approach. We review evidence that beneath the staggering complexity of human syntax, there is an extensive layer of nonproductive, nonhierarchical syntax that can be fruitfully compared to animal call combinations. This is the essential groundwork that must be explored and integrated before we can elucidate, with sufficient precision, what exactly made it possible for human language to explode its syntactic capacity, transitioning from simple nonproductive combinations to the unrivalled complexity that we now have.

A growing body of evidence suggests that animals are capable of combining independently meaningful vocalizations together into larger structures with a derived or transparent meaning [1–10]. In a recent critique, Bolhuis and colleagues [11] challenge these findings, primarily the idea that these data are comparable with compositional syntactic structures in human language and their potential relevance for understanding the evolution of language. Specifically, the authors assert that, in comparison to animal communication systems, syntax in language is based on fundamentally different organisational principles, which severely limits the prospects of successful comparisons across species. Here, we address the concern raised by Bolhuis and colleagues and outline why we think it is not only possible to directly compare human and animal call combinations but also why it is important to do so.

Human language is no doubt unique in the complexity of its expressions. One of the most striking aspects of this is that we command a great variety of syntactic configurations, such as modification (*a* *true* *biologist*), coordination (*biologists* *and* *linguists*), or predication (*the linguist* *objected*), and that these configurations can stack in a way that creates exceedingly complex dependencies (as in *neither*_*1*_ [*did the linguist*_*2*_ [*we consulted*] *object*_*2*_] *nor*_*1*_ [*was she*_*3*_ *interested*_*3*_], in which dependencies are indicated by subscripts). How did the capacity for this dazzling complexity emerge in humans? This question remains one of the major challenges in the field of language evolution [12, 13].

In answering the question, Bolhuis and colleagues start from what is known as the Minimalist Program [11, 14]. This research program seeks to reduce any kind of syntax in human language to a single computational operation of recursive combination, termed merge. Basically, merge just combines two elements (e.g., *the* and *apples*) to form a single set (*the apples*). However, what makes the operation really powerful is that it can be applied recursively to its own output or input. When merge is reapplied to its output, this generates simple hierarchical structure: e.g., merging *ate* with *the apples* yields the hierarchical structure *ate* [*the apples*]. When merge is reapplied to its input, it yields complex dependencies: e.g., reapplying merge to the element *what* from an already-merged structure [*John ate what*] yields *what* [*John ate what*], a structure that is argued to underlie complex dependencies as in *guess what*_1_ *John ate*_1_ in which *what* must refer to the object of *ate*. An immediate consequence of this approach is that, despite initial appearances, there are no genuine cases of nonhierarchical combinations in any human language. merge is the only relevant operation, hierarchical and recursive by nature—the essence of human syntax.

Reducing human syntax to a single operation is parsimonious and therefore an evolutionary scenario worth exploring. Indeed, if this reduction were successful, the consequences for evolutionary biology would be wide reaching. Firstly, unravelling the origin of syntax would reduce to understanding the evolutionary origin of merge. Secondly, it would render the comparative approach, a key method in evolutionary biology, obsolete: Even if one takes the simplest combinations in human language (such as *duck and cover*!), according to the Minimalist Program, they will be generated by the same merge operation as the most complex ones. Any observed similarity between the simplest combinations in animals and humans would therefore be deceptive. Unless nonhuman species can also be shown to master the most complex combinations present in language, it must be assumed that their simplest combinations are not generated by merge either. This makes any comparison a futile exercise [15, 16]. This is, in a nutshell, the idea from which Bolhuis and colleagues’ objections stem.

While potentially attractive and certainly interesting, merge is a thesis [17], not an observed fact that is established by convergent evidence. Moreover, it is just one of a plethora of theories linguists are currently exploring to account for language’s syntactic complexity [18]. It therefore seems worthwhile, if we are to fully understand the evolutionary origins of syntax, to consider alternative theoretical approaches and compare their empirical potential. In particular, it seems advisable to keep open the avenues paved by the classical toolbox of evolutionary biology, whereby a complex trait is decomposed into its component parts, each of which can then be independently investigated and subsequently used to reconstruct a trait’s evolution step by step.

In the case of human syntax, a common decomposition distinguishes layers or degrees of complexity [19, 20], thereby rejecting the reduction of these layers to a single operation such as merge. From a comparative perspective, two decompositions are particularly promising: (1) simple syntax that is limited to nonhierarchical combinations versus complex syntax that allows hierarchical, potentially recursive combinations; and (2) nonproductive syntax with fixed, one-off combinations versus productive syntax with unlimited combinations. Below, we address each of these in turn.

Consider a case of simple syntax such as *duck and cover*! It is not hierarchical, as the two commands have the same status in the sentence. But the combination still involves syntax, not just loose adjacency in discourse (unlike Bolhuis and colleagues’ example, *how are you // I’m feeling good* [11]). The evidence comes from the (fairly arbitrary) constructional constraints that languages impose on such combinations [21, 22]. E.g., in English, we can’t usually leave out *and* and still keep *duck* and *cover* under a single intonation curve (‘duck-cover’, parallel to the idiomatic *go eat*!); other languages allow this (or even lack an equivalent of *and* entirely). At the same time, English *and* can be used to demarcate conceptual groups [23, 24], each with the same syntactic status (*run and watch*, *and cover*! versus *run*, *watch*, *and cover*!); other languages have different means for this [25]. Further, in English, we can use *and* to join either verbs or nouns (e.g., *tea and coffee* next to *duck and cover*); other languages use different conjunctions here. Finally, English *and* usually imposes a linear order that reflects event order; other languages have special constructions that escape this [26]. What these and other constructional constraints show is that combinations like *duck and cover* are perfectly well part of syntax, yet they lack hierarchical structure. Note that under the Minimalist Program, however, even in these cases, a hierarchical analysis is imposed because apart from occasional exceptions [27–29], this approach does not recognize nonhierarchical, *n*-ary branching structures to begin with [30]. But there are many linguistic theories that do not impose a hierarchy (e.g., [24, 31–34]), and hierarchical analyses of *and* combinations are well known to incur empirical problems [29, 35].

Aside from productive combinations, hierarchical or not, human syntax also involves prefabricated—idiomatic and formulaic—combinations like *gimme a break*, *under attack*, or *green as grass* [36], forming a well-known *pain in the neck* [37] for computational approaches. Prefabricated expressions of this kind make up a store of knowledge comparable in size to the number of words we know [38] and characterize between about 25% and 50% of the phrases we use in conversation (depending on the context) [39]. Similarly, within words, next to productive markers such as *-ed*, which can even be added to a word invented on the spot (*she’s wooked*), there are many unproductive markers such as *-en* that are limited to prefabricated words (as in *she’s eaten*). While prefabrication triggers special effects in language processing, there is compelling experimental evidence that the brain’s production [40–43] and comprehension [44–47] systems nevertheless recognize these expressions as combinations with a compositional structure. Likewise, during language learning, children [48–51] and adults [52] are found to treat irregular, prefabricated expressions as internally structured, e.g., when generalizing patterns.

What do these observations entail for evolutionary biology? One important implication is that, underlying the vast complexity of human syntax, there exists a nontrivial layer of simple and nonproductive combinations. Given that many animals have been shown to also produce such simple nonproductive structures, we argue that the comparative approach once again becomes relevant, and useful comparisons between animals and humans can and should be made. Importantly, the comparisons need to be specific, not generic (Fig 1): it is useful to compare combinations that link alarm calls or mobbing calls (*Danger*, *come here*!) in animals with command coordination (*Duck and cover*!) in humans, but it is obviously less instructive to compare them with more complex structures that involve both coordination and modification in a single expression (such as Bolhuis and colleagues’ example *old men and women*, where *men and women* is a coordination structure and *old* adds a modification structure, either to *men* alone or to *men and women* together [11]). Both species and human languages greatly differ in the range of syntactic combinations they allow [53, 54], and wholesale comparisons are not helpful.

**Fig 1:**
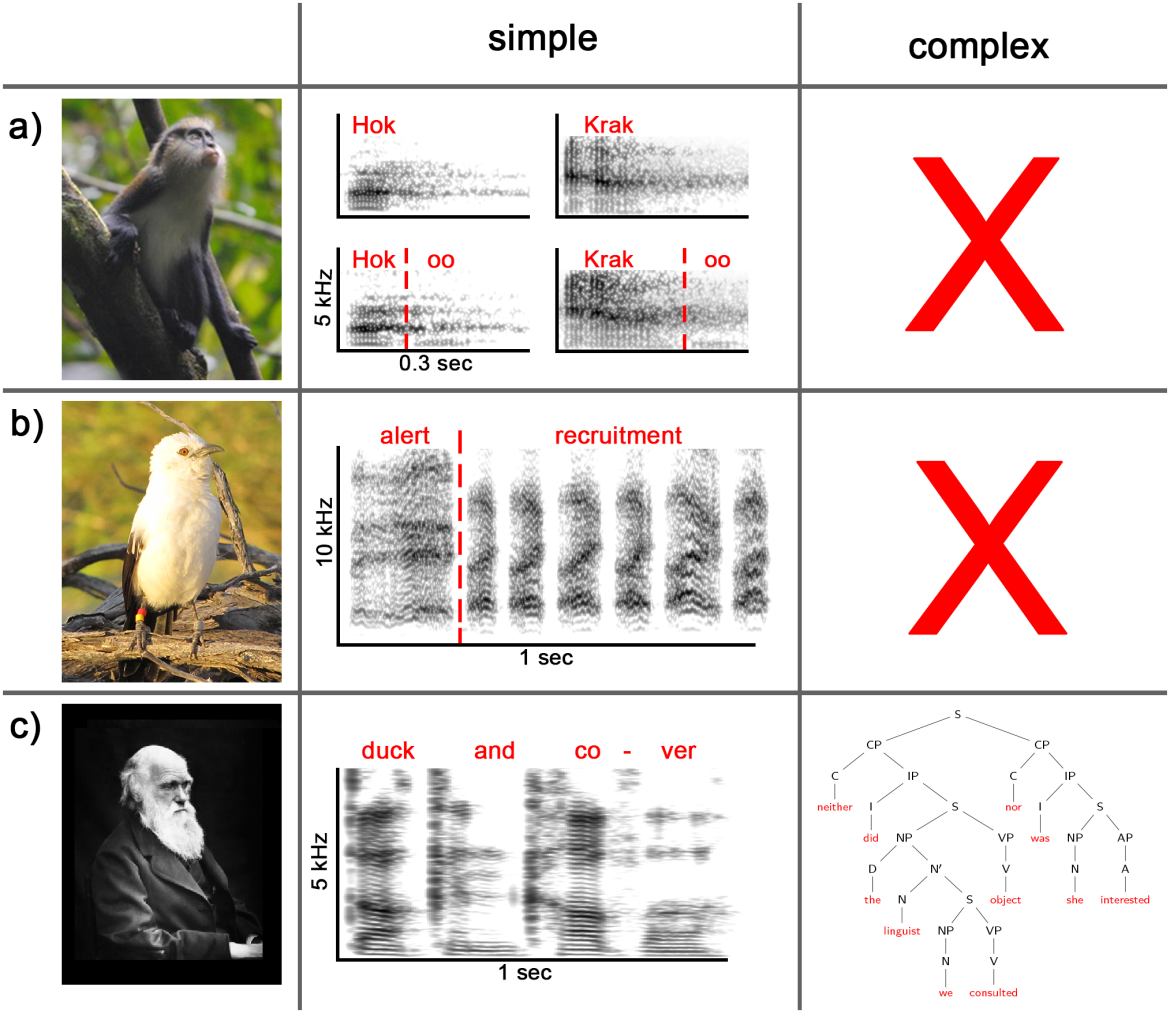
Simple and complex examples of compositionality in animals and humans. a) Compositionality in primates: Male Campbell’s monkeys produce ‘krak’ alarms (to leopards) and ‘hok’ alarms (to eagles), but both calls can also be merged with an ‘-oo’ suffix to generate ‘krak-oo’ (to a range of disturbances) and ‘hok-oo’ (to non-ground disturbances) [55]. In playback experiments, suffixation has shown to be meaningful to listeners [5], suggesting that it is an evolved communication function. This system may qualify as limited compositionality, as the meanings of krak-oo and hok-oo are directly derived from the meanings of krak/hok plus the meaning of—oo [56]. Spectrograms regenerated using data from [55]. b) Compositionality in birds: Pied babblers produce ‘alert’ calls in response to unexpected but low-urgency threats and ‘recruitment’ calls when recruiting conspecifics to new foraging sites [6, 57]. When encountering a terrestrial threat that requires recruiting group members (in the form of mobbing), pied babblers combine the two calls into a larger structure, and playback experiments have indicated that receivers process the call combination compositionally by linking the meaning of the independent parts [6]. c) Compositionality in humans: humans are capable of producing both simple, nonhierarchical compositions (e.g., ‘*Duck and cover*!’) and complex hierarchical compositions and dependencies. Photo in panel A credited to Erin Kane. Photo in panel B credited to Sabrina Engesser. A, adjective; AP, adjective phrase; C, conjunction; CP, conjunction phrase; D, determiner; I, Inflection-bearing element; IP, inflectional phrase; N, (pro-)noun; NP, noun phrase; S, sentence; V, verb; VP, verb phrase.

Whether or not comparable combinations across species are indeed evolutionarily related is an unresolved issue. It is an empirical question that requires a thorough understanding of the range of constructional constraints acting on human languages and of how communicators understand, recognize, and processes these combinations, both simple and complex, productive and nonproductive. The same understanding is needed for animal call combinations. Only once these data are at hand can we feasibly start to empirically explore the phylogeny of syntax, e.g., whether alarm call combinations in monkeys are truly homologous to a conjunction of commands in humans or whether a bird alert–recruitment call combination is a genuine analogue of monkey alarm combinations or of human command conjunctions.

The foundations for this research have been laid. Research on animal call combinations has been making rapid progress ever since it started to focus on constructional constraints [1–3, 5–8, 10, 55–63], just like in human syntax: E.g., which combinations are possible? Does ordering matter? Is the acoustic transition between elements stable or variable? Is the meaning broad or narrow?

Answering such questions is laborious and involves numerous small steps of comparison. However, we submit that it is essential groundwork that must be carried out before we can elucidate, with sufficient precision, what exactly made it possible for human language to explode its syntactic capacity, transitioning from simple, nonproductive combinations to the unrivalled complexity that we now have. Perhaps it was a wholesale replacement of all this by a single operation merge, as Bolhuis and colleagues propose. Perhaps it was the addition of computational resources to handle dependencies in more complex hierarchies [64] or the evolution of asymmetrical structure in syntax so that we understand ‘animal syntax’ as a kind of syntax and not as a kind of animal [65]. We don’t know, and there certainly exist many more theoretical options. We propose that resolving these fundamental questions empirically requires a detailed point-by-point comparison of the combinations and the constructional constraints that animals and humans impose on their expressions. Broad verdicts along the lines of ‘like humans’ versus ‘unlike humans’ are as unhelpful in language evolution as in any other domain of evolutionary biology.
